# Isoliquiritigenin inhibits the proliferation, migration and metastasis of Hep3B cells via suppressing cyclin D1 and PI3K/AKT pathway

**DOI:** 10.1042/BSR20192727

**Published:** 2020-01-02

**Authors:** Yun Huang, Chen Liu, Wu-Cha Zeng, Guo-Yan Xu, Jian-Min Wu, Zhi-Wen Li, Xuan-Yu Huang, Rong-Jin Lin, Xi Shi

**Affiliations:** 1 Depatment of General Medicine, The First Affiliated Hospital of Fujian Medical University, Fuzhou 350004, China; 2 Department of Geriatric Medicine, The First Affiliated Hospital of Fujian Medical University, Fuzhou 350004, China; 3 Department of Cadre’s Ward, The First Affiliated Hospital of Fujian Medical University, Fuzhou 350004, China; 4 The First Department of Chemotherapy, The First Affiliated Hospital of Fujian Medical University, Fuzhou 350004, China; 5 Department of Neurology, The First Affiliated Hospital of Fujian Medical University, Fuzhou 350004, China; 6 Department of Neurology, Fujian Medical University Union Hospital, Fuzhou 350004, China; 7 Depatment of Nursing, The First Affiliated Hospital of Fujian Medical University, Fuzhou 350004, China

**Keywords:** cyclin D1, hepatocellular carcinoma, Isoliquiritigenin, PI3K/AKT

## Abstract

The overall survival rate of patients with hepatocellular carcinoma (HCC) has remained unchanged over the last several decades. Therefore, novel drugs and therapies are required for HCC treatment. Isoliquiritigenin (ISL), a natural flavonoid predominantly isolated from the traditional Chinese medicine Glycyrrhizae Radix (Licorice), has a high anticancer potential and broad application value in various cancers. Here, we aimed to investigate the anticancer role of ISL in the HCC cell line Hep3B. Functional analysis revealed that ISL inhibited the proliferation of Hep3B cells by causing G_1_/S cell cycle arrest *in vitro*. Meanwhile, the inhibitory effect of ISL on proliferation was also observed *in vivo*. Further analysis revealed that ISL could suppress the migration and metastasis of Hep3B cells *in vitro* and *in vivo*. Mechanistic analysis revealed that ISL inhibited cyclin D1 and up-regulated the proteins P21, P27 that negatively regulate the cell cycle. Furthermore, ISL induced apoptosis while inhibiting cell cycle transition. In addition, phosphatidylinositol 3′-kinase/protein kinase B (PI3K/AKT) signal pathway was suppressed by ISL treatment, and the epithelial marker E-cadherin was up-regulated when the mesenchymal markers Vimentin and N-cadherin were down-regulated. In brief, our findings suggest that ISL could be a promising agent for preventing HCC tumorigenesis and metastasis.

## Introduction

Hepatocellular carcinoma (HCC) is considered as one of the most aggressive cancers with a low postoperative survival rate and high recurrence rate, and the 5-year survival rate is less than 20% [1,2]. At present, surgical resection, radiotherapy, and chemotherapy are the main treatment methods used for HCC in clinical settings. However, the overall survival rate of patients with HCC has remained unchanged over the last several decades [2]. Patients had a poor prognosis mainly owing to tumor recurrence and distant metastasis. Therefore, it is very important to develop novel effective drugs for HCC treatment.

Glycyrrhizae Radix (Licorice) has been used in traditional Chinese medicine over thousands of years. Isoliquiritigenin [2′,4′,4-trihydroxychalcone (ISL)], a natural flavonoid predominantly isolated from the licorice root, was considered to be the main bioactive ingredient. ISL exerts various pharmacological actions such as anti-viral, anti-microbial, anti-inflammatory, anti-oxidative, and immunomodulatory effects [3–6]. It also exhibits anti-tumorigenic activities by inhibiting proliferation and angiogenesis, inducing apoptosis, causing cell cycle arrest, or obstructing metastasis in various types of cancer [7,8]. A wide spectrum of tumors has been demonstrated to be sensitive to ISL, including breast tumors, human cervical tumors, ovarian tumors, human melanoma, lung tumors, and glioma [3]. Previous reports have demonstrated that ISL regulates the progression of human HCC cells by regulating activation of MAPK/STAT3/NF-Κb, JAK1/STAT1, IRF3/MyD88, et al [9–11]. However, the anti-tumor effects of ISL on HCC are not fully characterized, especially in *in vivo* experiments.

The capability of sustained uncontrollable proliferation is a major feature of cancer cells. This feature is closely related to abnormalities of cell cycle regulation and mechanisms of apoptosis resistance [12–14]. Inhibiting the transition of cell cycle and inducing apoptosis of tumor cells have become a mature strategy and research direction for anti-tumor therapy, especially in HCC treatment [12,15,16]. Therefore, the influence of ISL on the cell cycle and apoptosis of HCC cells is worthy of research.

In the present study, we sought to verify the effects of ISL on the proliferation, migration, and metastasis of the HCC cell line Hep3B *in vitro* and *in vivo*. Further, we attempted to search for suitable molecular mechanisms to explain the abovementioned functional changes. Our results revealed that ISL could potentially be exploited as a natural and an efficient adjuvant in HCC treatment.

## Materials and methods

### Reagents

ISL (Herbpurify; Chengdu, China) was dissolved in dimethyl sulfoxide (DMSO; Sigma–Aldrich; St. Louis, MO) to a 20-mM stock solution, stored at -20°C, and serial dilutions were made in distilled water. Other reagents are described below.

### Cell culture

Normal human hepatocyte cell line LO2 and human HCC cell line Hep3B were purchased from the American Type Culture Collection (Manassas, VA). The cells were cultured in Dulbecco’s modified Eagle’s medium (DMEM; GIBCO BRL, Life Technologies, Carlsbad, CA) with 10% FBS (GIBCO BRL, Life Technologies, Carlsbad, CA) at 37°C in a 5% CO_2_ incubator.

### Xenograft model, pulmonary metastasis model, and treatments

Female BALB/c-nu/nu mice (18–20 g) aged 4–5 weeks were provided by Shanghai Laboratory Animal Center (Shanghai, China). Mice were housed under a sterile environment and maintained under a daily 12-h light/dark cycle, which was controlled by qualified staff at the Fujian Medical University Laboratory Animal Center, Fuzhou, China. The mice were put to death by cervical dislocation. All procedures were conducted with animal welfare considerations, and the study protocol was approved by the animal experiment ethics committee of the Fujian Medical University. Two different mouse models were used to observe the *in vivo* effect of ISL on HCC cells.

The subcutaneous model was constructed as follows: Hep3B cells (2.0 × 10^6^ cells) were suspended in 100-ml serum-free DMEM, and the mixture was injected into the flank of nude mice. Ten days after the cells were injected, when tumors were observable, mice were randomly separated into two groups (*n*=5) and were injected intraperitoneally with ISL (50 mg/kg) or vehicle once everyday for 3 weeks. At the end of the treatment, tumors were collected and their weight and volume were measured prior to fixation (volume (cm^3^) = length (cm) × width^2^ (cm^2^)/2). Some of the tumor tissues were fixed in 4% paraformaldehyde for Hematoxylin and Eosin (H&E) or immunohistochemical staining. The others were stored in −80°C until lysed for Western blotting analysis.

Pulmonary metastasis model was designed as follows: Hep3B cells were injected at a concentration of 2.0 × 10 ^6^ through the tail vein in each nude mouse (*n*=5). After 1 week, mice were randomly separated into test and control groups and were injected intraperitoneally with ISL (50 mg/kg) or vehicle once everyday for 5 weeks. At the end of the 6th week, all mice were killed and the lungs were removed.

### Cell viability and proliferation assay

MTT assay was used to test the viability and proliferation of Hep3B cells. Cells were implanted in 96-well plates at a specific density (1500 cells/well for proliferation assay; 5000 cells/well for viability assay) and cultivated for 1 or 2 days. After ISL stimulation (according to the specific experiment), 20 μl of 3-(4,5-dimethylthiazol-2-yl)-2,5-diphenyltetrazolium bromide (MTT) (5 mg/ml; Sigma, St. Louis, U.S.A.) was added to each well. After incubation for 4 h, we removed the culture medium containing MTT and added 150 μl DMSO into each well. The optical density (OD) value of each well was measured at 490 nm.

### Colony formation assa*y*

Cells were implanted on to six-well culture plates at 200 cells/well. After being cultivated for 14 days, cells were washed twice with phosphate buffer saline (pH 7.4) and dyed with 2.0% Crystal Violet. A microscope was used to make sure each colony contained ≥50 cells. All assays were independently performed thrice.

### EdU proliferation assay

Apollo488 *In Vitro* Imaging Kit (RiboBio, Guangzhou, China) was used according to the manufacturer’s protocol. Briefly, cells were incubated with 10 μM EdU for 2 h before fixation with 4% paraformaldehyde, permeabilization with 0.3% Triton X-100, and stained with EdU. Cell nuclei were stained with 5 μg/ml DAPI (4′,6-diamidino-2-phenylindole) for 5 min. The number of Edu-positive cells was counted under a microscope in five random fields (200×). All assays were independently performed thrice.

### Scratch-wound healing assay

After ISL stimulation, cells were seeded into six-well plates. When the cells became completely attached, the cell layer was gently scratched over a straight line, and then the cells were washed with phosphate buffer saline (pH 7.4); furthermore, 2 ml maintenance medium (DMEM with 2% FBS) was added to the cell mixture and the cells were observed under a microscope (200×) at the same point on the line at different time points (0, 48 h).

### Cell migration assay

Transwell assays were performed to evaluate cell migration. Cell migration assay was performed using cell culture inserts (Corning, New York, U.S.A.). Briefly, cells (1 × 10^5^ cells/200 μl in a serum-reduced medium) were placed in the upper chamber of a transwell apparatus, while the bottom chambers were filled with 500 μl DMEM supplemented with 10% FBS. Cells were incubated at 37°C for 24 h. At the termination of the incubation period, the migrant cells on the lower surface of the membranes were fixed and stained with 2.0% Crystal Violet. Microphotographs of five different fields were obtained, and the cells were counted.

### RNA isolation and quantitative real-time polymerase chain reaction

Total RNA was extracted from Hep3B cells using TRIzol (Takara, Shiga, Japan). One microgram of total RNA was reverse transcribed into cDNA. Real-time (RT) PCR was performed to analyze the genes of interest by employing specific primers and SYBR-Green as a fluorescent dye (Bio-Rad Laboratories, Hercules, CA, U.S.A.). The following primers were used: cyclin D1 (forward: GATCAAGTGTGACCCGGACTG; reverse: AAAATGCTCCGGAGAGGAGG), GAPDH (forward: CTGCACCACCAACTGCTTAG; reverse: GTCTTCTGGGTGGCAGTGAT). Experiments were performed according to the manufacturer’s instructions (Takara, Shiga, Japan). All experiments were performed thrice.

### Western blotting

The protein expression in tumor tissues or Hep3B cells was detected by Western blot. Total protein extracts were obtained by centrifugation at 15000×***g*** at 4°C for 15 min and the protein concentrations were quantified using a BCA protein assay kit (Pierce; Thermo Fisher Scientific, Inc). Equal amounts of cell lysates (20 μg) were separated by 10% SDS/polyacrylamide gel electrophoresis and transferred to PDVF membranes. After blocking with 5% skim milk at room temperature for 2 h, cells were incubated with the indicated primary antibodies. The primary antibodies included cyclin D1 (#55506), p27 (#3686), p21 (#2947), PI3K (#4257), p-PI3K (Tyr^458^, #17366), AKT (#4685), p-AKT (Ser^473^, #4060), Vimentin (#5741), E-cadherin (#14472), N-cadherin (#4061), cleaved-Caspase-3 (Asp^175^, #9661), cleaved-caspase-9 (Asp^330^, #52873), Bcl-2 (#3498), Bax (#2772), cleaved-PARP (Asp^214^, #5625) antibodies (1:1000; Cell Signaling Technology, Danvers, MA, U.S.A.), p-PI3K antibody (#11508, 1:1000; Signalway Antibody LLC, Maryland, U.S.A.) and GAPDH antibody (60004-1-Ig, 1:7500; Proteintech, Rosemont, U.S.A.). Following overnight incubation at 4°C, membranes were washed three times with 0.1% Tween 20 in TBS and incubated with secondary antibodies. The secondary antibodies were donkey anti-mouse and goat anti-rabbit (1:7500; LI-COR Biosciences, Lincoln, NE). Protein bands were detected using a chemiluminescent HRP detection kit (Millipore, Billerica, MA). All experiments were performed thrice.

### Flow cytometric analysis of the cell cycle

Cell cycle analysis was performed using Cell Cycle and Apoptosis Analysis Kit (Beyotime, Beijing, China). Briefly, the cultured cells were collected and digested and fixed in cold 70% ethanol and stored overnight at 4°C. Ethanol was subsequently removed by centrifugation (1000×***g*** for 5 min), followed by staining with Propidium Iodide and RNase A for 30 min at 37°C in the dark. The cell cycle was analyzed using flow cytometry (BD LSRFortessa™, X-20; BD Biosciences, San Jose, CA).

### Histopathology and immunohistochemistry

Fixed tumor tissues were processed for paraffin embedding and were sectioned into 4-µm sections. The sections were stained with H&E according to standard protocols. For immunohistochemical staining, the tumor tissues slides were incubated with diluted primary antibodies against anti-Ki67 antibody (ab15580) (Abcam, Cambridge, U.K.) according to the manufacturer’s instructions. The primary antibody was detected using biotinylated goat anti-rabbit antibody followed by 3,3-diaminobenzidine in the kit (Univbio, Shanghai, China).

### Serum measurements

Alanine aminotransferase (ALT) and aspartate aminotransferase (AST) were measured in serum using biochemical kits (Nanjing Jiancheng Bioengineering Institute, Nanjing, China) according to manufacturer’s instructions.

### Statistics

Data were expressed as means ± standard deviation. Statistical analysis of two groups was performed by employing a two-tailed Student’s *t* test. Two or more groups were statistically analyzed using the ANOVA test by employing SPSS 22.0 software. *P*-values of <0.05 were considered to be statistically significant.

## Results

### ISL exhibits greater cytotoxic effects on Hep3B cells than on normal hepatocyte

**Figure 1:**
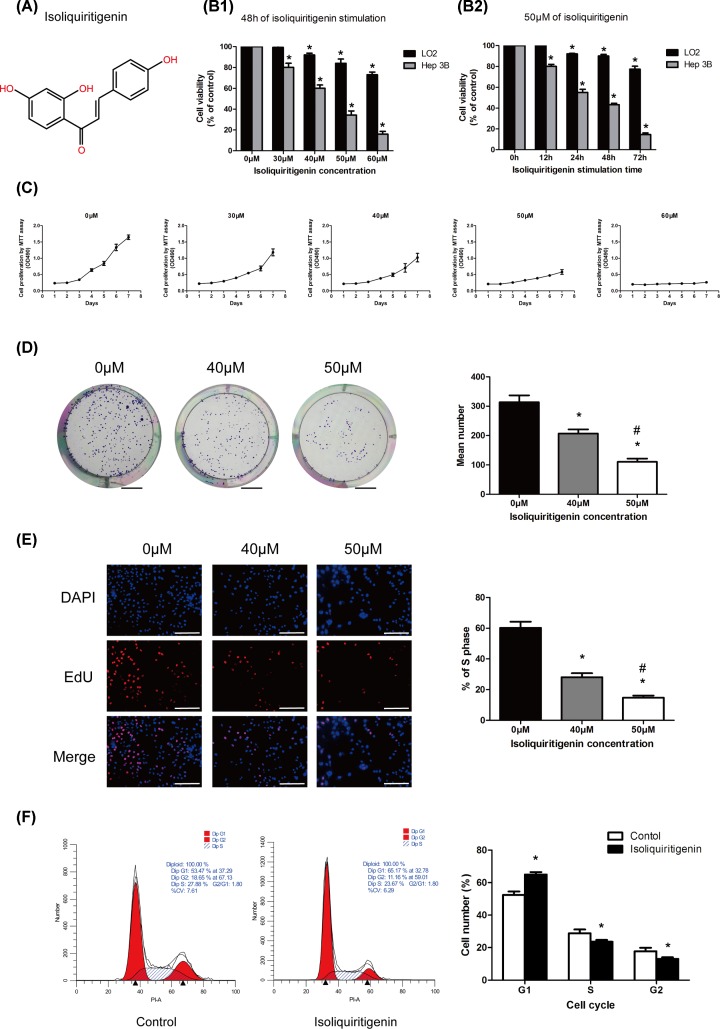
ISL inhibited proliferation of Hep3B cells (**A**) Chemical structure of ISL. (**B**) Normal human hepatocyte cell line (LO_2_) and HCC cell line (Hep3B) were treated with various concentrations of ISL for 48 h or incubated with 50 μM of ISLin for different times (12, 24, 48, and 72 h). Cell viability was measured using an MTT assay. (**C**–**E**) Hep3B cells were treated with varying concentrations of ISL for 48 h. Effect of ISL on proliferation function of Hep3B was analyzed by MTT (C), colony-forming assay (D), and EdU incorporation assay (**E**). (**F**) Cell cycle distribution analysis by flow cytometry. Hep3B cells were incubated with indicated 50 µM ISL for 48 h. All experiments were performed in triplicate. Data are presented as mean ± SEM. *P<0.05 relative to 0 μM of ISL concentration treatment group or control; ^#^*P*<0.05 versus 40 μM of ISL concentration treatment group. Scale bar: (D) 6 mm; (E) 200 μm.

The chemical structure of ISL is shown in Figure 1A. To determine the cytotoxic effects of ISL on the HCC cell line, Hep3B, and on human normal immortalized hepatocytes, LO2, we subjected both cell lines to ISL stimulation under different concentrations or stimulation durations and used MTT assay to observe the cell survival rate. The results showed that ISL is obviously cytotoxic to Hep3B cells with 50% inhibitory concentrations (IC_50_) of 42.84 ± 2.01 μM, and this effect is concentration- and time-dependent (Figure 1B). However, under the same ISL stimulus intensity, the survival ratio of LO2 cells was significantly less affected compared with Hep3B cells (IC_50_ > 60 μM). This result indicated that ISL exerts a greater cytotoxic effect on HCC cells than on normal hepatocytes.

### ISL inhibits the proliferation ability of HCC cells *in vitro*

Subsequently, we used Hep3B cell to investigate the effect of ISL on the proliferation capacity of HCC cells. Consistent with observations of cell cytotoxicity, MTT (Figure 1C), colony formation (Figure 1D), and EdU incorporation assays (Figure 1E) confirmed that ISL stimulation inhibited the proliferation of Hep3B cell and had an exact concentration-dependent effect. Furthermore, compared with the control group, we observed that the treatment of ISL (50 μM, 48 h) inhibited cell cycle transition from G_1_ to S phase in Hep3B cells (Figure 1F), which further explained the mechanism that ISL inhibits cell proliferation and suggested that ISL may cause changes in cell cycle-associated proteins.

### ISL suppresses the migration ability of HCC cells *in vitro*

**Figure 2:**
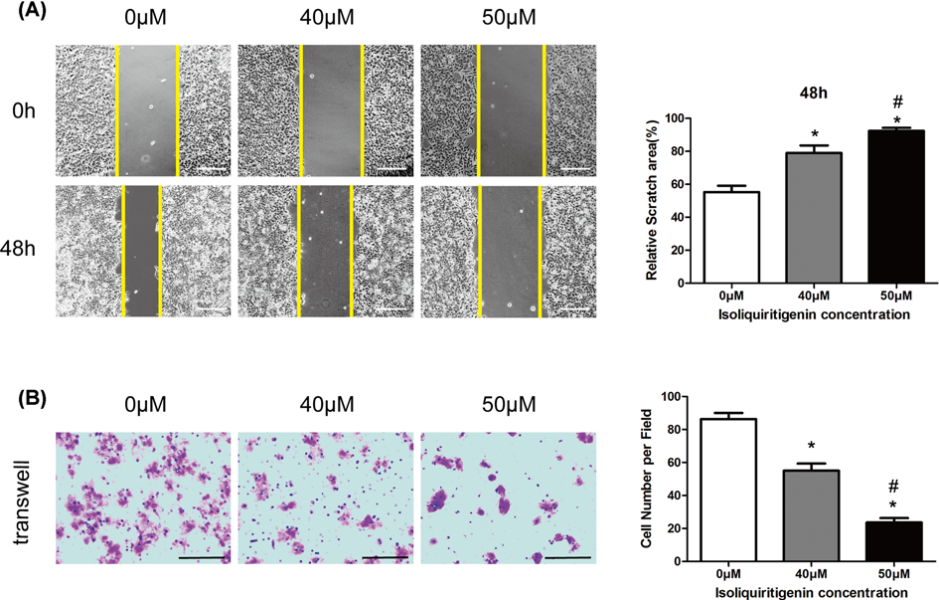
ISL inhibited migration of Hep3B cells The migration ability of Hep3B cells with or without ISL (40, 50 μM) stimulation for 48 h was detected by wound-healing assays (**A**) and transwell assay (**B**). All experiments were performed in triplicate. Data are presented as mean ± SEM. *P<0.05 relative to 0 μM concentration of ISL treatment group; ^#^*P*<0.05 versus 40 μM concentration of ISL treatment group. Scale bar: (A) 250 μm; (B) 200 μm.

The poor prognosis of HCC was associated with HCC cell migration capacity. Therefore, we investigated the effect of ISL on Hep3B cell migration capacity. Hep3B cells were stimulated with different concentrations of ISL (40, 50 μM) for 48 h before performing further experiments. Wound-healing assay showed that the migration of Hep3B cells in the ISL-treated group was decreased compared with that in the control group, showing a concentration-dependent trend (Figure 2A). Similarly, a transwell assay reconfirmed the inhibitory effect of ISL on Hep3B cells migration. ISL reduced the number of migration cells in a concentration-dependent manner (Figure 2B). These results revealed that ISL has the potential to inhibit HCC cell migration *in vitro*.

### ISL inhibits proliferation and metastasis of Hep3B cells *in vivo*

**Figure 3:**
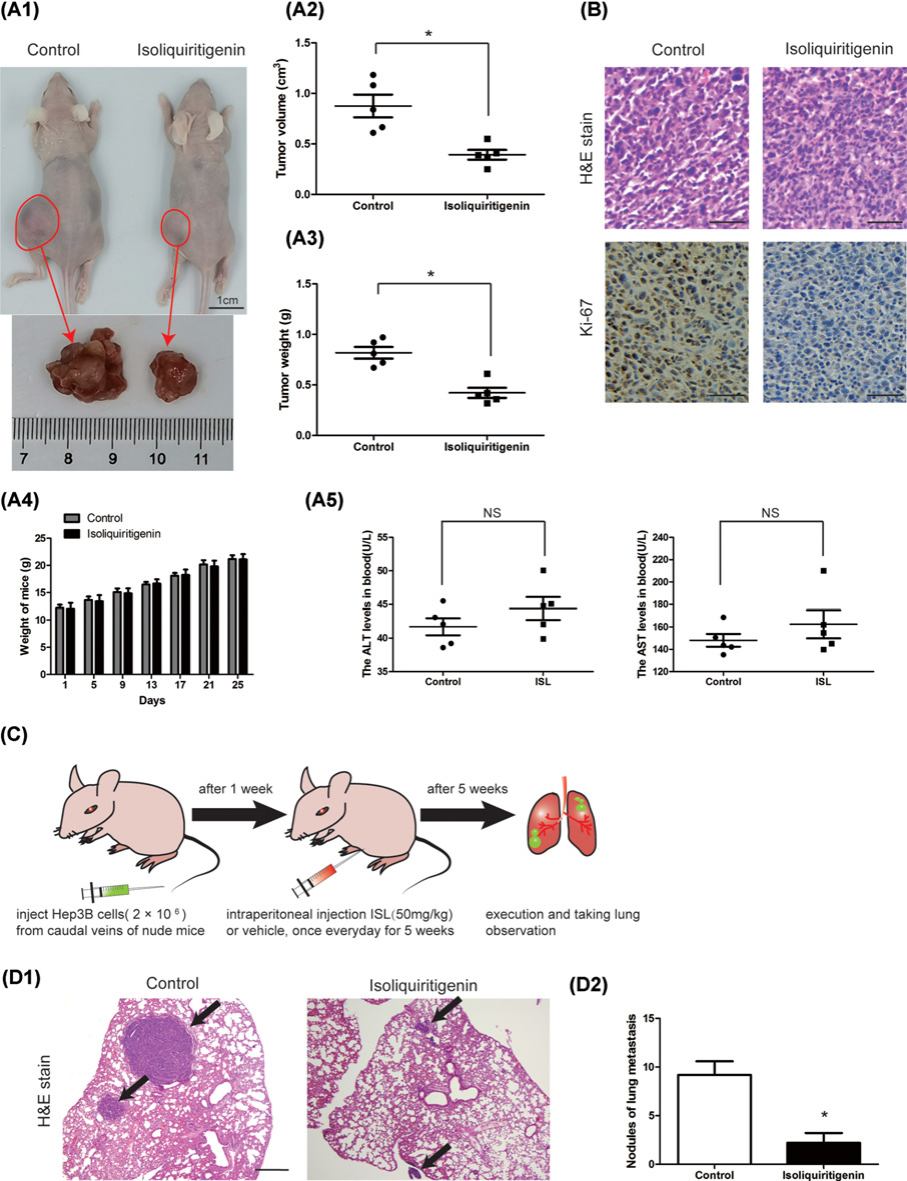
ISL inhibited proliferation and metastatasis of Hep3B cells *in vivo* (**A,B**) Subcutaneous injection Hep3B cells plus ISL treatment in nude mice inhibited tumor growth. (**A1**) The photos of tumors isolated from killed nude mice of the indicated groups. The volume (**A2**) and weight (**A3**) of the tumors. (**A4**) The body weight of ISL–treated group or control group mice. (**A5**) Serum ALT, AST levels. (B) Representative pictures of tumors’ sections stained with H&E and Immunohistochemistry (Ki-67). (**C**) The pulmonary metastasis model was adopted to evaluate the effect of ISL on metastasis in Hep3B cells. (**D1**) Representative pictures of pulmonary metastatic nodules with H&E staining. (**D2**) The statistics of pulmonary metastatic nodules. Scale bar: (A1) 1 cm; (B) 50 μm; (D1) 500 μm. *P<0.05 versus control.

To further determine the inhibitory effects of ISL on HCC cell proliferation, we constructed a subcutaneous tumor formation model using Hep3B cell line and nude mice. Compared with the control group, ISL-treated mice had a reduced tumor burden, in terms of both the average tumor size or xenograft weight (Figure 3A1–A3). The expression level of Ki67, a protein that marks cell division and proliferation activity, was reduced in the ISL-treated group relative to that in the control group (Figure 3B). Therefore, ISL treatment inhibited the growth of the xenograft. It is noteworthy that no statistical difference was observed with regard to changes in mice weight between the treatment and control groups, there also was no significant difference in blood ALT and AST levels between the two groups, suggesting that ISL has mild adverse effect *in vivo* (Figure 3A4,A5).

Furthermore, we constructed a pulmonary metastasis model using Hep3B cells to evaluate the effects of ISL on the metastatic growth of HCC cells (Figure 3C). H&E staining results revealed that the ISL-treated mice displayed a lower number and smaller size of lung metastases than that in the control group (Figure 3D1,D2). These results revealed that ISL has the potential to inhibit HCC cell proliferation and metastasis *in vivo*.

### ISL reduces the level of cyclin D1 and inhibits phosphatidylinositol 3′-kinase/protein kinase B signaling pathway

**Figure 4:**
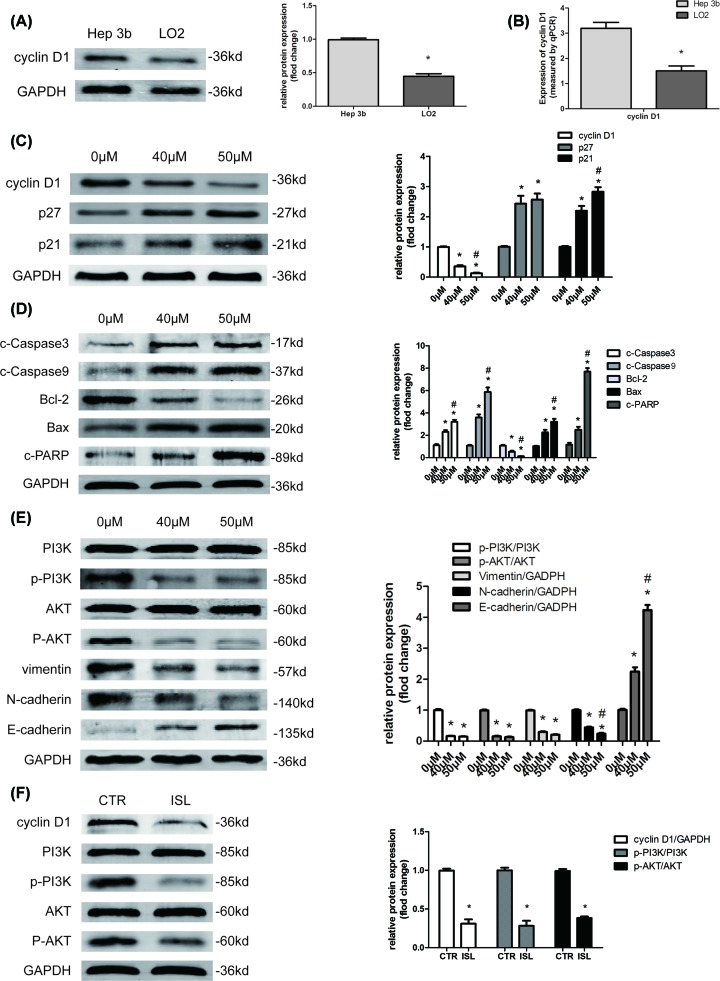
Effect of ISL on the cyclin D1 and phosphatidylinositol 3′-kinase/protein kinase B signal pathway (**A,B**) The expression of cyclin D1 was measured by Western blot analysis (A) and real-time PCR (B) in Hep3B and LO2 cells. (**C**–**E**) Hep3B cells were treated with ISL (40, 50 μM) for 48 h. (C) The protein levels of cyclin D1, p21 and p27 were assayed by Western blot. (D) The protein levels of cleaved-caspase-3, cleaved-caspase-9, Bcl-2, Bax, and cleaved-PARP were assayed by Western blot. (E) The protein levels of PI3K, p-PI3K, AKT, p-AKT, vimentin, E-cadherin, and N-cadherin were assayed by Western blot. (**F**) Xenograft tumors isolated from control and ISL-treated mice. Protein levels of cyclin D1, PI3K, p-PI3K, AKT, and p-AKT were assessed by Western blot in xenograft tumors. All experiments were performed in triplicate. Data are presented as mean ± SEM. *P<0.05 relative to 0 μM concentration of ISL treatment group or Hep3B group; ^#^*P*<0.05 versus 40 μM concentration of ISL treatment group.

Cyclin D1 belongs to the highly conserved cyclin family. The activity of cyclin D1 is required for cell cycle G_1_/S transition [17]. Given that ISL can affect the cell cycle and inhibit the transition from G_1_ to S cell cycle phase in Hep3B cells, we speculated that ISL may inhibit Hep3B cells proliferation by regulating the expression of cyclin D1. Compared with normal human hepatocyte LO2, the protein and mRNA levels of cyclin D1 were elevated in Hep3B cells (Figure 4A,B). This result suggests that the overexpression of cyclin D1 may be positively correlated with the malignancy of hepatoma cells. Further research revealed that after 48 h ISL stimulation, cyclin D1 expression in Hep3B cells was down-regulated in a dose-dependent manner (Figure 4C). Meanwhile, Western blot confirmed that Hep3B cells treated with ISL showed an up-regulation in the protein levels of the cell cycle negative regulatory protein, including p21 and p27 (Figure 4C). These results confirmed that ISL may inhibit HCC cell proliferation by inhibiting the cell cycle transition. Furthermore, considering that P21 and P27 can simultaneously regulate cell cycle transition and apoptosis [18,19], we further examined changes in apoptosis-related proteins. As shown in Figure 4D, after ISL treatment, the expression level of cleaved-caspase-3, cleaved-caspase-9, Bax, and cleaved-PARP were increased while Bcl-2 was decreased. These results suggested that ISL may induce apoptosis while inhibiting cell cycle transition, which further explained the molecular mechanism by which ISL inhibits cell proliferation.

Phosphatidylinositol 3′-kinase/protein kinase B (PI3K/AKT) pathway, a classical oncogenic signaling pathway, was reported to be closely related to ISL treatment [20,21]. As shown by the data, we observed that ISL suppressed the phosphorylated levels of PI3K and AKT proteins in Hep3B cells (Figure 4D). This indicated that ISL could inhibit PI3K/AKT signal pathway in HCC cells. These changes further affected the expression of epithelial-to-mesenchymal transition (EMT)-related protein including Vimentin, E-cadherin, and N-cadherin. With the weakening of the PI3K/AKT signal, the expression of the epithelial marker, E-cadherin, was up-regulated, whereas that of the mesenchymal markers, N-cadherin and Vimentin were inhibited (Figure 4E); this finding suggested that ISL could suppress EMT in HCC cells.

At the same time, we examined the levels of cyclinD1 and PI3K/AKT signaling in xenograft tumors isolated from control and ISL-treated mice. Similarly, in ISL-treatment group the xenograft showed a decrease in cyclin D1 protein levels and inhibition of PI3K/AKT signaling pathway (Figure 4F). The above results re-verified the therapeutic mechanism of ISL from *in vivo*.

## Discussion

In the present study, it was demonstrated that ISL suppressed the proliferation ability of the HCC cell line Hep3B *in vitro*, and this inhibitory effect is associated with the inhibition of G_1_/S transition of the cell cycle. Furthermore, our data demonstrated that ISL can inhibit the migration of Hep3B cells *in vitro*. For the first time, we performed tumorigenesis and metastasis assays using two different xenograft mouse models bearing tumors originating from Hep3B cells. The results of the *in vivo* experiment were consistent with those of the *in vitro* experiments, which further confirmed the effects of ISL on anti-tumor proliferation and metastasis. In conclusion, ISL may serve as a promising agent for HCC treatment, consistent with the findings from previous studies [22,23].

Cell cycle control involves multiple signal cascade transmission mechanisms including DNA replication, cell division, and proliferation [23]. G_1_, S, and G_2_/M work as three key checkpoints and strictly modulate the progression of the entire cell cycle. Disturbance in the checkpoints resulted in uncontrolled cell division and hyperproliferation in cancer cells [24]. Among them, the imbalance in cyclin-dependent kinases (CDKs) and CDK inhibitors (CDKIs), which are responsible for promoting and inhibiting cell cycle progression, respectively, plays a key role in tumor cell cycle disorder [25]. Cyclin D1, a member of the cyclin family (D1, D2, and D3) which functions as a regulatory subunit and forms a complex with CDK4 or CDK6 for regulating cell cycle transition from the G_1_ to S phase [26], is frequently overexpressed in cancer cells and dysregulates CDK activity. P21 and P27 have been reported as classic CDKIs, and negatively correlated with CDK4/6 and cyclin D1 expression [27,28]. In tumor cells, overexpression of cyclin D1 and down-regulation or dysfunction of its negative regulatory proteins P21 and P27 are very common, these changes are closely related to the abnormal proliferation of tumors. Molecular mechanisms leading to this abnormal expression include overexpression of oncogenes such as c-Myc [29], FBXO22 [30], miR-95-3p [31], deletion or mutation of tumor suppressor gene such as P53 [32], and abnormal activation of the cancer-promoting signaling pathways such as PI3K/AKT pathway [32–34]. In the present study, we noted an abnormal overexpression of cyclin D1 in Hep3B cells both at the mRNA and protein levels. ISL treatment down-regulated cyclin D1 and up-regulated the CDK inhibitory proteins P21 and P27. The above mentioned observations are consistent with those noted in previous studies involving inhibition of tumor cell proliferation by ISL [35]; this study further verified that ISL is a natural cyclin D1 inhibitor that suppresses HCC tumorigenesis by inducing G_1_/S phase arrest.

Apoptosis induction is closely related to cell cycle arrest. It was reported that following G_1_-phase cell cycle arrest, cells may enter the apoptotic pathway [36]. ISL is a natural apoptosis inducer in many cancers [20,23,37], and has been demonstrated can induce apoptosis in HCC cell HepG2 [38]. Interestingly, we found that apoptosis-related protein changes can also be observed in Hep3B cells after ISL treatment, including cleavage of caspase-9 and -3, and PARP; down-regulation of anti-apoptotic proteins such as Bcl-2 and up-regulation of pro-apoptotic protein Bax. These results suggest that ISL may induce apoptosis through P21/P27-dependent pathways.

The high mortality rate and low cure rate of cancer are, in large part, owing to the distant metastasis of advanced tumor, especially for HCC [1]. In the present study, we proved that ISL inhibits migration of Hep3B cells *in vitro* by cell wound healing assay and transwell assays. Consistent with the results *in vitro, in vivo* results revealed that ISL inhibited lung metastasis in Hep3B cells. These conclusions are similar to those from previous studies [39,40]. Although the detailed molecular mechanism of tumor metastasis has not yet been elucidated, EMT is considered to play a vital role in it and is widely and deeply studied in the fields of tumor progression, metastasis, and drug resistance [41,42]. Therefore, we speculate that ISL inhibition of migration and metastasis in Hep3B cells may be related to EMT inhibition. In the present study, the results of the Western blot experiment validated our hypothesis. After ISL treatment, the epithelial markers, E-cadherin, was up-regulated, whereas the mesenchymal markers, Vimentin (a cytoskeletal protein) and N-cadherin (a cell surface protein), were down-regulated. For the first time, we proved that ISL inhibits tumor migration and metastasis by inhibiting EMT in HCC cells.

In previous studies, ISL was reported to be involved in the regulation of the PI3K/AKT pathway, which is a classic crucial oncogenic signaling pathway [20,43]. Activation of the PI3K/AKT signaling pathway affects multiple pivotal aspects of tumor development, including cell-cycle transition, proliferation, cell adhesion, motility, and invasiveness [44,45]. Abnormal activation of the PI3K pathway is also prevalent in HCC, characterized by abnormally elevated levels of phosphorylation [46]. Numerous previous studies have shown that ISL inhibits the PI3K/AKT pathway by reducing the level of related protein phosphorylation [20,35,39]. Our Western blot analysis results were similar to those from previous studies. Interestingly, the PI3K/AKT pathway also participated in the positive regulation of EMT and cyclin D1 [26,33]. Therefore, the suppression of PI3K/AKT pathway by ISL in our study more strongly supports the results from previous researches, and it also proves that ISL can play a role as a natural PI3K inhibitor in HCC treatment.

Our study indirectly demonstrates the safety of ISL treatment for normal hepatocytes by comparing the difference in survival ratio between Hep3B cells and the normal hepatocytes LO2 at the same time point of ISL stimulation or concentration of ISL. Furthermore, ISL treatment had little negative effect on the growth of mice. At the same time, there was no significant difference in hematological parameters (ALT, AST) between the ISL treatment group and the control group. These conclusions supported that ISL may be used as a natural, effective, and safe cyclin D1 and PI3K/AKT inhibitor in HCC therapy. However, further studies are needed to validate the *in vivo* investigations for the targetorgan toxicity or side effects.

In summary, our data first demonstrated that ISL suppresses the cell cycle transition and inhibits the proliferation and migration of Hep3B cells *in vivo* and *in vitro* by suppressing cyclin D1 and PI3K/AKT pathway. Consequently, these results broaden our understanding of the mechanisms underlying ISL effects in HCC and reinforce its potential anticancer property as a popular Chinese herbal medicine (CHM).

## Abbreviations

ALT: alanine aminotransferase
AST: aspartate aminotransferase
Bax: Bcl-2 associated X protein
Bcl-2: B-cell lymphoma-2
CDK: cyclin-dependent kinase
CDKI: CDK inhibitor
DMEM: Dulbecco’s modified Eagle’s medium
DMSO: dimethyl sulfoxide
EdU: 5-ethynyl-2′-deoxyuridine
EMT: epithelial-to-mesenchymal transition
FBXO22: F-box only protein 22
GAPDH: glyceraldehyde-3-phosphate dehydrogenase
HCC: hepatocellular carcinoma
H&E: Hematoxylin and Eosin
IC_50_: 50% inhibitory concentration
IRF3: interferon regulatory factor3
ISL: isoliquiritigenin
MTT: 3-(4,5-dimethylthiazol-2-yl)-2,5-diphenyltetrazolium bromide
MyD88: myeloid differentiation factor88
NF-kB: nuclear factor kappa-B
PARP: poly ADP-ribose polymerase
PI3K/AKT: phosphatidylinositol 3′-kinase/protein kinase B
